# Magnetic Properties and the Electronic Structure of the Gd_0.4_Tb_0.6_Co_2_ Compound

**DOI:** 10.3390/ma13235481

**Published:** 2020-12-01

**Authors:** Marcin Sikora, Anna Bajorek, Artur Chrobak, Józef Deniszczyk, Grzegorz Ziółkowski, Grażyna Chełkowska

**Affiliations:** 1 Institute of Physics, University of Silesia in Katowice, 75 Pułku Piechoty 1A, 41-500 Chorzów, Poland; marcin.y.y@gmail.com (M.S.); anna.bajorek@us.edu.pl (A.B.); artur.chrobak@us.edu.pl (A.C.); grzegorz.ziolkowski@us.edu.pl (G.Z.); 2 Institute of Materials Engineering, University of Silesia in Katowice, 75 Pułku Piechoty 1A, 41-500 Chorzów, Poland; jozef.deniszczyk@us.edu.pl

**Keywords:** magnetic properties, rare earth–transition metal compounds, magnetocaloric effect, electronic structure

## Abstract

We report on the comprehensive experimental and theoretical studies of magnetic and electronic structural properties of the Gd_0.4_Tb_0.6_Co_2_ compound crystallization in the cubic Laves phase (C15). We present new results and compare them to those reported earlier. The magnetic study was completed with electronic structure investigations. Based on magnetic isotherms, magnetic entropy change (Δ*S_M_*) was determined for many values of the magnetic field change (Δ*μ*_0_*H*), which varied from 0.1 to 7 T. In each case, the Δ*S_M_* had a maximum around room temperature. The analysis of Arrott plots supplemented by a study of temperature dependency of Landau coefficients revealed that the compound undergoes a magnetic phase transition of the second type. From the *M*(*T*) dependency, the exchange integrals between rare-earth R-R (*J_RR_*), R-Co (*J_RCo_*), and Co-Co (*J_CoCo_*) atoms were evaluated within the mean-field theory approach. The electronic structure was determined using the X-ray photoelectron spectroscopy (XPS) method as well as by calculations using the density functional theory (DFT) based Full Potential Linearized Augmented Plane Waves (FP-LAPW) method. The comparison of results of ab initio calculations with the experimental data indicates that near *T_C_* the XPS spectrum collects excitations of electrons from Co*3d* states with different values of exchange splitting. The values of the magnetic moment on Co atoms determined from magnetic measurements, estimated from the XPS spectra, and results from ab initio calculations are quantitatively consistent.

## 1. Introduction

Magnetic properties of Laves phase compounds have recently been of great interest to researchers [1,2,3,4]. A relatively simple crystal structure, facilitating the interpretation of the investigation results, was the main reason for this. In this family of materials, special attention should be paid to Laves phases containing rare-earth (R) and transition metal (T) atoms. Conventional representatives of this class of compounds are the RCo_2_, the magnetic properties of which strongly depend on the kind of rare earth metal involved in the alloy. The RCo_2_ compounds with non-magnetic R-ions such as Y or Lu show enhanced Pauli paramagnetism and undergo a metamagnetic transition. Under the influence of an external magnetic field exceeding a certain critical *H_c_* value, a transition from a paramagnetic to a ferromagnetic state occurs [5] accompanied by the increase of initially negligible magnetic moment on the Co site even by 0.5 *μ_B_*. [6,7]. In the RCo_2_ compounds with magnetic R ions, in the ordered state, the molecular field *H_mol_* may exceed the critical field required to induce the metamagnetic transition in the *d*-electron subsystem [5,8]. The molecular field enhanced by the external magnetic field can easily exceed the critical value *Hc*, resulting in an increase of the magnetic moment of Co up to a value of about 1 *μ_B_* [5,8,9]. It is known that RCo_2_ compounds show a magnetic structure with parallel or antiparallel alignments of the magnetic moments of R and Co ions, for light or heavy R elements, respectively [8]. Substitution of other kinds of atoms with RCo_2_ results in a new family of compounds called pseudo-binaries like the R(Co_1−x_T_x_)_2_, the R_1-y_R’_y_Co_2_ or even four-component R_1−y_R’_y_(Co_1−x_T_x_)_2_ compounds. They are particularly interesting because of additional interactions between magnetic moments (4f’-4f, 4f-3d, 4f’-3d, and others) which may significantly alter the magnetic properties of the original material but also strongly modify their electronic structure. RT_2_ intermetallics are also known as materials exhibiting a significant magnetocaloric effect (MCE) [10]. The value of MCE in these compounds usually has its maximum around the Curie temperature (*T_C_*) and can be quite high in the case of the first-order phase transition (FOPT). The magnetic materials for which *T_C_* is located near room temperature are particularly attractive due to the possibility of using them for magnetic refrigeration in consumer devices. [11]. This technology of cooling is more efficient than gas compression refrigeration [12]. MC materials can also be applied in passive cooling processes to control heating in power conversion applications [13] as well as in medicine, e.g., in the treatment of malignant tumors by the method of hyperthermia [14].

Among the materials with *T_C_* near room temperature, there is one composition (x = 0.6) in the Gd_1−x_Tb_x_Co_2_ series studied by Zhou et al. [15], where the MCE was determined only for Δ*μ*_0_*H* = 2 T. In our study, we focused on more detailed and extended magnetic investigations of the Gd_0.4_Tb_0.6_Co_2_ compound, for many values of the magnetic field change (Δ*μ*_0_*H*) varying from 0.1 to 7 T, and in a broader temperature range. Besides the determination of the magnetic entropy change Δ*S_M,_* we additionally calculated the relative cooling power parameter RCP and the refrigeration capacity, RC. Considering the Gd_0.4_Tb_0.6_Co_2_ as a basis for the new class of multi-component compounds, we have studied its electronic structure, particularly in the valence band range, which is essential for magnetic properties. The study of the electronic structure was conducted experimentally using XPS (X-ray Photoelectron Spectroscopy), and theoretically by ab initio calculations. Our results are completely new for the compound under investigation. Moreover, using the two-sublattice model, in the mean-field theory (MFT) approximation, the exchange integrals *J_RR_*, *J_RCo_*, and *J_CoCo_* were evaluated. Within this theory, we determined the magnetic moment of Co atoms *μ_Co-MFT_* and compared it with ts values obtained by other methods.

## 2. Experimental and Computational Details

The Gd_0.4_Tb_0.6_Co_2_ samples were prepared by the arc melting method from high-purity elements (99.99% purity) under an argon atmosphere. Excess amounts of 1 wt % of gadolinium and terbium were added to overcome weight losses during the melting. To obtain a high homogeneity of compounds, the samples were re-melted several times. Afterwards, the as-cast samples were wrapped in tantalum foil, placed in a quartz tube, and annealed at 800 °C for two weeks. The crystal structure was determined by the X-ray diffraction technique (XRD) using an Empyrean PANalytical diffractometer. The measurements were performed at room temperature with Cu K_α_ source and 2θ changing from 15 to 140 degrees. All magnetic measurements were carried out using a SQUID magnetometer MPMS XL–7 (Quantum Design Inc., San Diego, CA, USA) in the temperature range from 2 to 400 K under a magnetic field of up to 7 T. The electronic structure of the investigated compound was studied using the XPS method. The XPS spectra were obtained with monochromatized Al K_α_ radiation (*hω* = 1486.6 eV) at room temperature using a PHI 5700/660 spectrometer (Physical Electronics Inc., Eden Prairie, MN, USA). All spectra were measured immediately after breaking the sample in the vacuum of 10^−9^ Torr. The breaking in the high vacuum resulted in clean surfaces free of oxygen and carbon contamination.

The electronic structure of Gd_0.4_Tb_0.6_Co_2_ was calculated employing the ab initio DFT based full-potential linearized augmented plane waves (FP-LAPW) method [16] using the WIEN2k package (WIEN2k_19.1, released 25 June 2019, Institute of Materials Chemistry, TU Wien, Vienna, Austria) [17]. The electronic states of constituent atoms are divided into core (strongly bound), valence band, and weakly bound core states described by local orbital functions [16]. Accordingly, the following electronic configurations were assumed: Tb–[Kr+4*d*^10^] {5*s*^2^5*p*^6^}_LO_(6*s*^2^4*f* ^9^)_VB_; Gd–[Kr+4*d*^10^]{5*s*^2^5*p*6}_LO_(6*s*^2^4*f* ^7^5*d*^1^)_VB_ and Co–[Ne]{3*s*^2^3*p*^6^}_LO_(3*d*^7^4*s*^2^)_VB_. For the core states, the fully relativistic DFT formalism was applied while the local orbitals (LO) and VB states were treated within the scalar relativistic approximation. For the LO and VB states, the spin-orbit (SO) interaction was taken into account using the second variational method [16]. A generalized gradient approximation for the exchange-correlation (XC) potential parametrized for solids (PBEsol) [18] was used. To account for the enhanced Coulomb correlation in the group of R4*f* states we have used the LDA + U formalism [19]. In the present study, we have taken an effective Coulomb correlation parameter U_eff_ equal to 6.5 eV and 7.1 eV for Tb [20] and Gd [21], respectively. The radii of muffin-tin spheres were assumed 0.1376 nm and 0.1217 nm for R and Co, respectively. The following values of parameters decisive for the accuracy of calculations employing WIEN2k code were assumed: *l*_max_ = 12, *G*_max_ = 14, and *K*_max_ = 9/*R*_MT_. The k mesh division 11 × 11 × 11 (714 k-vectors in the IBZ) used in calculations was tested to ensure a total energy convergence of 0.01 eV/fu. For simulations of the fractional concentration of R atoms in Gd_0.4_Tb_0.6_Co_2_ a conventional unit cell comprising eight formula units of TbCo_2_ was employed basing on which the superstructures were prepared in which three Tb atoms were replaced by the Gd ones.

## 3. Results and Discussion

### 3.1. Crystal Structure

**Figure 1:**
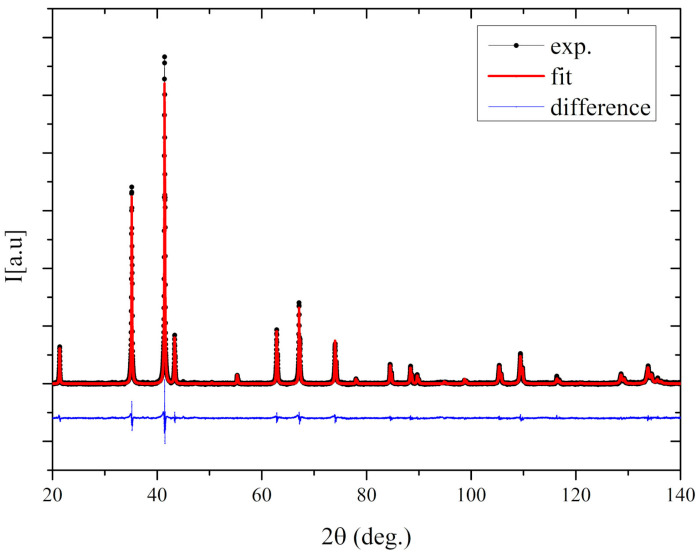
The X-ray diffraction (XRD) patterns for the Gd_0.4_Tb_0.6_Co_2_ compound.

Figure 1 presents the X-ray diffraction patterns of the investigated compound. The crystal structure was refined using the Rietveld method, and the analysis was carried out using the non-commercial Maud software [22,23]. The analysis showed that the Gd_0.4_Tb_0.6_Co_2_ crystallized in the MgCu_2_ type of structure (Fd-3m space group). The sample was found homogeneous and free of other phases. The refined cell parameter was equal to 7.2563 ± 0.0010 Å, slightly larger than that reported by Zhou [15] (7.2285 Å).

### 3.2. Magnetic and Magnetocaloric Properties

**Figure 2:**
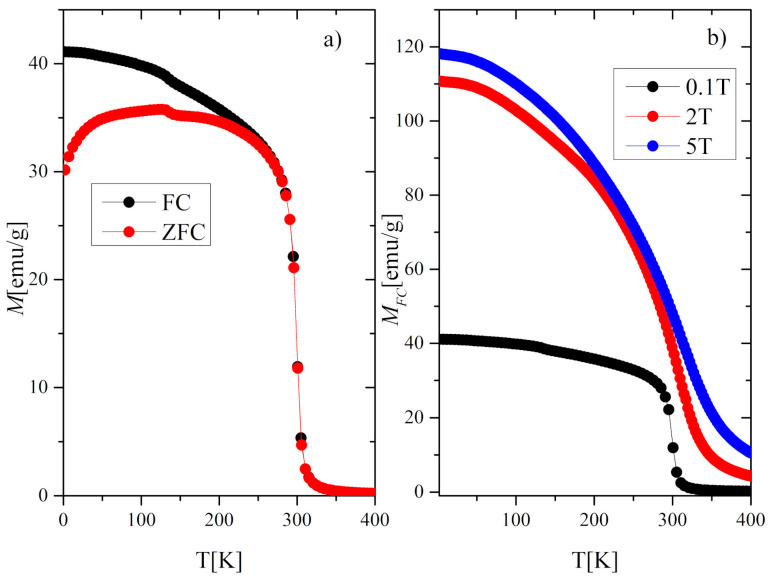
(**a**) Magnetization *M* versus temperature in the FC and ZFC mode at a magnetic field of 0.1 T, (**b**) Magnetization in the FC mode at 0.1, 2, and 5 T.

**Figure 3:**
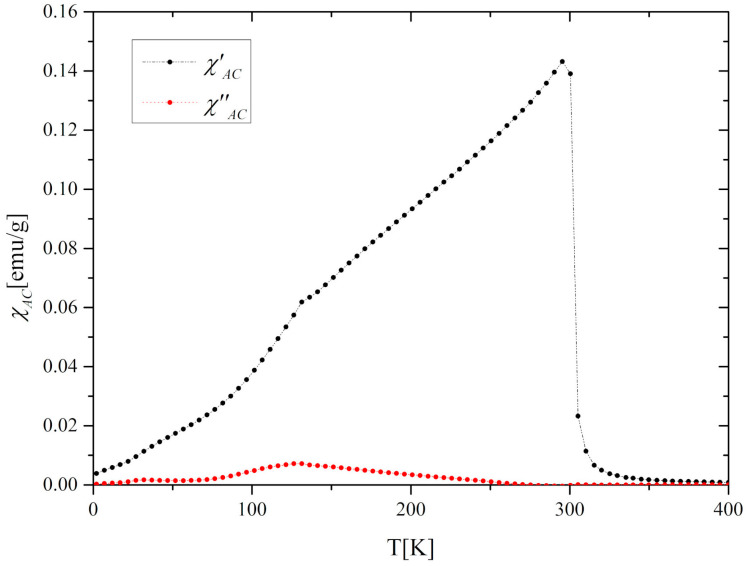
The *AC* magnetic susceptibility (*χ_AC_*) of the Gd_0.4_Tb_0.6_Co_2_ compound.

The temperature dependence of the magnetization *M(T)* of the Gd_0.4_Tb_0.6_Co_2_ compound in zero-field cooling (ZFC) and field cooling (FC) modes at the external magnetic field of 0.1T is presented in Figure 2a. These thermomagnetic curves exhibit irreversible behavior in the low-temperature range, which often happens in this kind of compounds [10,24]. The magnetic ordering temperature *T_C_* estimated from the minimum value of *dM/dT* was equal to 300.7 K in both ZFC and FC modes. A peculiarity which is visible in Figure 2a at 136 K may be attributed to the partial reorientation of the Tb spin toward the easy axis direction. The effect is also reflected in *AC* magnetic susceptibility *χ_AC_* at the same temperature (Figure 3). The application of a higher magnetic field of 2T and 5T caused the disappearance of this characteristic feature (Figure 2b). A similar effect was observed for TbNi_2_, where it was additionally confirmed by the elastic neutron diffraction [25].

**Figure 4:**
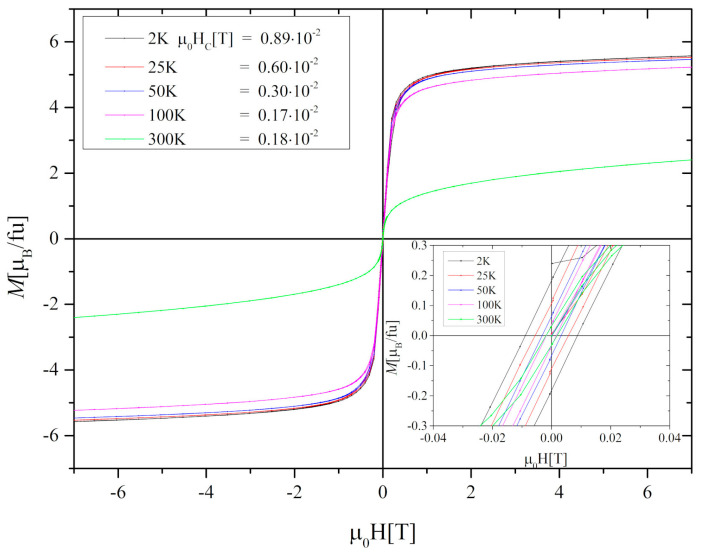
Hysteresis loops of the Gd_0.4_Tb_0.6_Co_2_ compound measured at 2 K, 25 K, 50 K, 100 K, and 300 K. The magnetization unit of [µ_B_/fu] was derived from [emu/g] using the formula: 1 [emu/g] = 0.1078 × 10^21^/N [µ_B_/fu], where N is the number of formula units of Gd_0.4_Tb_0.6_Co_2_ in 1 g.

The hysteresis loops measured at 2 K, 25 K, 50 K, 100 K, and 300 K show minimal hysteresis losses with the value of the coercive field decreasing from 0.89 × 10^−2^ T to 0.18 × 10^−2^ T at 2 K and 300 K, respectively (Figure 4). Moreover, no saturation has been found even at *μ*_0_*H* = 7 T.

**Figure 5:**
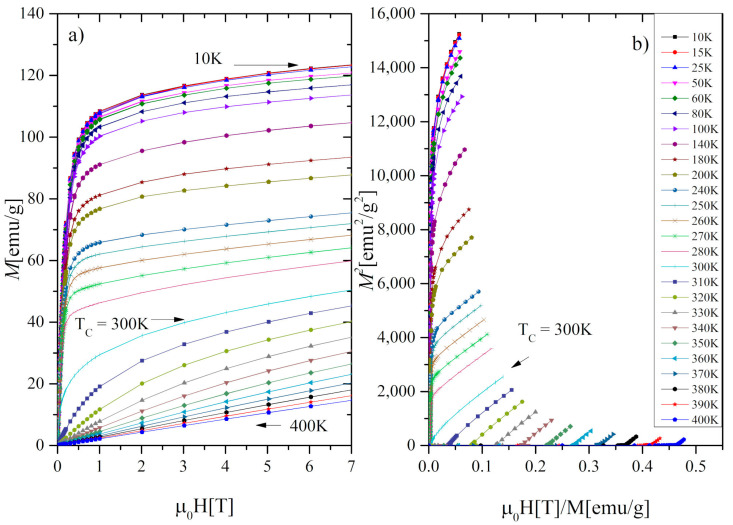
(**a**) Magnetic isotherms and (**b**) Arrott plots for the Gd_0.4_Tb_0.6_Co_2_.

Using *M(H)* data at 2 K, the saturation magnetization (*μ_S_*) was determined from extrapolation to zero of 1/*H* in the 1/*H* vs. *M* dependence. The procedure gave *μ_S_* *=* 5.85*μ_B_*. Assuming that the total magnetic moment of Gd_0.4_Tb_0.6_Co_2_ is given by *μ_S_ =* 0.4*μ_Gd_ +* 0.6*μ_Tb_ +* 2*μ_Co_* and taking *μ_Gd_ =* 7*μ_B_*, *μ_Tb_ =* 9*μ_B,_* (calculated using the formula: $\mu_{R}=g_{R}\mu_{B}J_{R}$, where $g_{R}$ is a Landé factor and $J_{R}$ is a ground state total angular momentum [26] we have got the value of the magnetic moment of the cobalt atom *μ_Co_* = −1.17*μ_B_.* The negative sign indicates the antiparallel alignment of the R and Co moments. Figure 5a shows the isothermal magnetization *M* as a function of the applied magnetic field of up to 7 T. The Arrott plots (Figure 5b) indicate that the investigated compound undergoes a phase transition of the second-order (SOPT). To confirm the observation we determine the type of phase transition using the Landau expression for the magnetic free energy (*F*) [10]:

(1) $$F=\;\frac{1}{2}a\left(T\right)M^{2}+\frac{1}{4}b\left(T\right)M^{4}+\frac{1}{6}c\left(T\right)M^{6}-\mu_{0}HM$$

The temperature dependence of Landau coefficients *a*(*T*), *b*(*T*), and *c*(*T*) is used to identify the type of the phase transition. They are accessible through the following relation between *M* and *H*:

(2) $$\mu_{0}H=a\left(T\right)M+b\left(T\right)M^{3}+c\left(T\right)M^{5}$$

Essentially, the order of the magnetic transition is governed by the sign of *b*(*T*). The first-order phase transition takes place if *b*(*T_C_) <* 0, while the second-order phase transition occurs when *b*(*T_C_)* ≥ 0.

**Figure 6:**
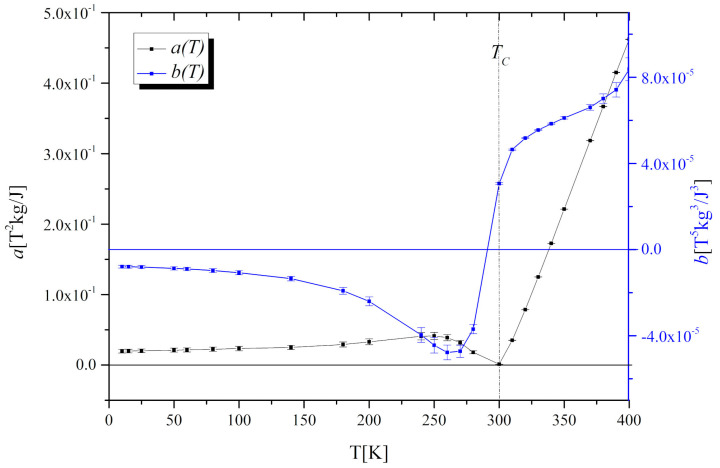
The temperature dependence of Landau coefficients *a*(*T*) and *b*(*T*) for the Gd_0.4_Tb_0.6_Co_2_.

The coefficients were determined by fitting the Equation (2) to magnetic isotherms *μ*_0_*H*(*M*) (Figure 5a). As it is visible in Figure 6 the *a*(*T*) exhibits a minimum in the vicinity of *T_C_* and the *b*(*T*) parameter is positive, which proves the second-order character of the phase transition, consistently with the conclusion based on our Arrott plots and in agreement with the results reported in [15].

It is known that in materials showing SOPT, the magnetic entropy change is lower than in those with FOPT; however, the SOPT materials usually have a broader working-temperature range which enhances their cooling efficiency (see Equation (4)), which is essential for potential applications, e.g., in magnetic refrigerators [27,28,29,30].

**Figure 7:**
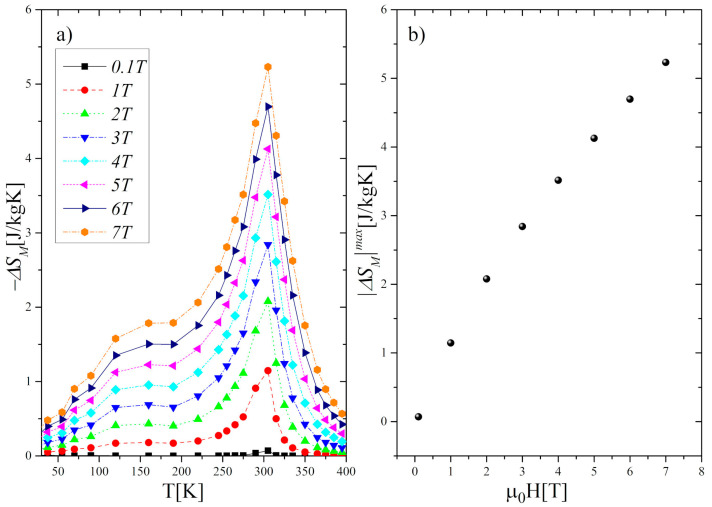
(**a**) Magnetic entropy changes (−Δ*S_M_*) in the Gd_0.4_Tb_0.6_Co_2_ as a function of temperature and magnetic field. (**b**) Variation of the maximum of the magnetic entropy changes |Δ*S_M_*|*^max^* with the growth of a magnetic field.

To calculate the magnetic entropy change Δ*S_M_* basing on the magnetic isotherms we used Maxwell’s relation:

(3) $$\Delta S_{M}\left(T,\Delta H=H_{1}-H_{0}\right)=\;\overset{H_{1}}{\underset{H_{0}}{\int }}{\left(\frac{\partial M\left(T,\;H\right)}{\partial T}\right)}_{H}dH$$

where *H*_0_ and *H*_1_ are the initial and final magnetic fields, respectively. The calculated values of the Δ*S_M_* as a function of temperature and magnetic field are presented in Figure 7.

As can be seen, the maxima of the entropy changes |Δ*S_M_*|*^max^* occur near *T_C_*, which is typical of compounds exhibiting SOPT. The value of |Δ*S_M_*|*^max^* obtained under magnetic field changes between 1 T and 7 T increased from 1.15 J/kgK to 5.23 J/kgK, respectively. Moreover, we found that the Δ*S_M_* curves are symmetric with respect to *T_C_* only in a limited temperature range (*T_C_* *±* 50 K). Both the height and width of the curves increase monotonically with the growth of the magnetic field. In effect, the value of *δT_FWHM_*, defined as the full-width at half-maximum of the |Δ*S_M_*| peak, increased from 34 K at Δ(*μ*_0_*H*) *=* 1 T to 64 K at Δ(*μ*_0_*H*) *=* 7 T. To assess the cooling efficiency, we calculated the relative cooling power parameter *RCP* using the formula:

(4) $$RCP=\;{\left|\Delta S_{M}\right|}^{max}\;\delta T_{FWHM}$$

The cooling efficiency was also evaluated using the value of refrigerant capacity (*RC*) defined as the amount of heat that can be transferred from the cold end (at *T_cold_*) to the hot end (at *T_hot_*):

(5) $$RC=\;\overset{T_{hot}}{\underset{T_{cold}}{\int }}{\left|\Delta S_{M}\right|}^{max}dT$$

The values of both parameters increased significantly with increasing magnetic field.

Thus, the RC parameter increased from 31.16 J/kg to 259.40 J/kg, whereas the RCP from 39.40 J/kg to 298.17 J/kg with a *μ*_0_*H* change from 1 T to 7 T.

### 3.3. MFT Analysis

To estimate the 3d–3d and 3d–4f exchange interactions in the Gd_0.4_Tb_0.6_Co_2_ compound, we considered its magnetic structure as consisting of two magnetic sublattices formed by R and Co moments. Making the assumption that magnetic moments of R and T(Co) atoms are directed oppositely, the magnetization *M* of the system can be treated as a superposition of *M*_R_ and *M*_T_ described by the formula:

*M* = |*M_R_* − *M_T_*| (6)

**Figure 8:**
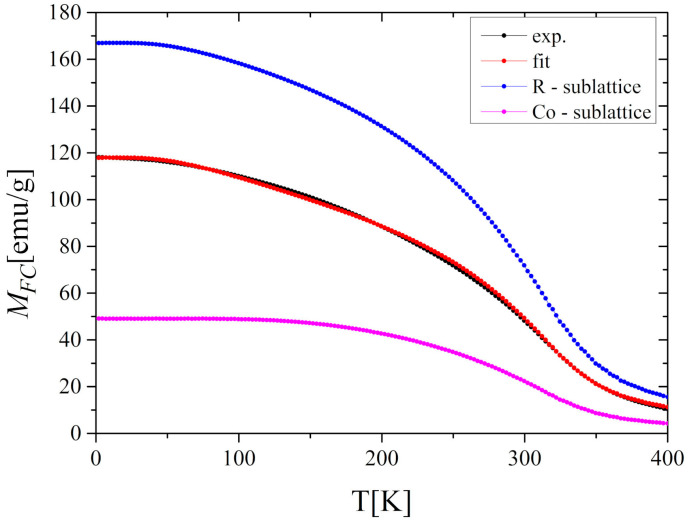
The *M_FC_*(*T*) dependence at *μ*_0_*H* = 5 T and the results of MFT analysis for the Gd_0.4_Tb_0.6_Co_2_.

In the MFT approximation, the *M_R_* and *M_T_* magnetizations can be described as follows [9,31,32,33]:

(7) $$\;M_{R}=-N_{R}\mu_{B}g_{R}\langle J_{R}\rangle =\;-N_{R}\mu_{B}g_{R}J_{R}B_{J_{R}}\left(\frac{g_{R}J_{R}H_{R}\mu_{B}}{k_{B}T}\right)$$

(8) $$M_{T}=-N_{R}\mu_{B}g_{T}\langle J_{T}\rangle =-N_{R}\mu_{B}g_{T}J_{T}B_{J_{T}}\left(\frac{g_{T}J_{T}H_{T}\mu_{B}}{k_{B}T}\right)$$

(9) $$\;H_{R}=\frac{2J_{RR}Z_{RR}{\left(g_{R}-1\right)}^{2}}{N_{R}g_{R}^{2}\mu_{B}^{2}}M_{R}+\frac{2J_{RT}Z_{RT}\left(g_{R}-1\right)\left(g_{T}-1\right)}{N_{T}g_{R}\mu_{B}^{2}}M_{T}+H_{ext}$$

(10) $$H_{T}=\frac{2J_{TR}Z_{TR}\left(g_{R}-1\right)\left(g_{T}-1\right)}{N_{R}g_{R}^{2}\mu_{B}^{2}}M_{R}+\frac{2J_{TT}Z_{TT}{\left(g_{T}-1\right)}^{2}}{N_{T}g_{T}^{2}\mu_{B}^{2}}M_{T}+H_{ext}$$

where *B* is the Brillouin function, *N_T_* and *N_R_* are the numbers of T and R atoms per unit volume, *μ_B_* is the Bohr magneton, *g* is the Landé factor, *J* is the total angular momentum, *H_R_*, *H_T_* refers to the magnetic field acting on the corresponding site, *H*_ext_ is an external magnetic field, and *k*_B_ is the Boltzmann constant. The exchange integrals *J_RR_*, *J_TT_*, and *J_RT_* (= *J_TR_*) can be determined by fitting the above equations to empirical thermomagnetic curves. For this purpose, we have used the *M(T)* dependence under a magnetic field of 5 T, which was strong enough to avoid the influence of domain effects and achieve a relatively high saturation (Figure 2b). Figure 8 compares the fitted curve with the experimental data and presents the results of the MFT analysis. Because the R sublattice consists of two kinds of rare-earth atoms (described by the Brillouin functions with different ground states) the presented analysis refers to an average value of R magnetic moment. Assuming that the average coordination numbers of R-R (Z_RR_), R-Co (Z_RCo_), Co-R (Z_CoR_), and Co-Co (Z_CoCo_) are equal: 4, 12, 6, and 6, respectively [33], we have obtained the following values of exchange coupling integrals: *J_RR_* = 1.9 × 10^−23^ J, *J_RCo_* = −1.17 × 10^−22^ J and *J_CoCo_* = 3.59 × 10^−22^ J.

It should be noted that the order of magnitude of *J_RCo_* is the same as that obtained by Duc [9] for GdCo_2_ and TbCo_2_ compounds (1.88 × 10^−22^ J and 1.81 × 10^−22^ J, respectively). The negative sign of *J_RCo_* means the antiparallel coupling between R and Co moments. Taking *μ_Gd_* = 7 *μ_B_* and *μ_Tb_* = 9 *μ_B_*, and following the above assumptions, it is also possible to determine the average magnetic moment per Co atom. As a result, we got *μ_Co-MFT_ = *−1.22 *μ_B_* which is very close to the value obtained from the magnetic measurements at 2 K (−1.17 *μ_B_*) and from ab initio calculations (−1.28).

## 4. X-ray Photoelectron Spectroscopy (XPS)

**Figure 9:**
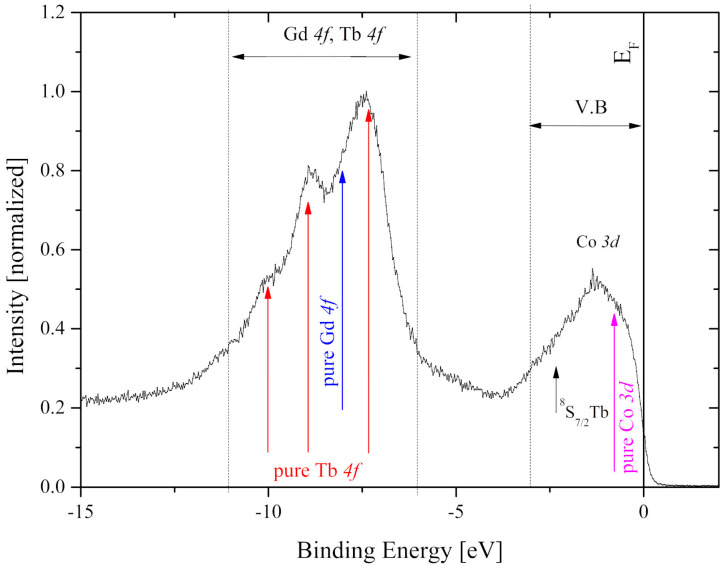
The XPS valence band of the Gd_0.4_Tb_0.6_Co_2_. The spectrum is normalized to the maximum intensity in this energy range. Vertical arrows show the positions of spectral lines observed in elemental Gd, Tb, and Co.

The valence band spectrum of the Gd_0.4_Tb_0.6_Co_2_ compound in the binding energy range from −15 eV to −2 eV is presented in Figure 9.

In pure metals, *4f* states are visible at −8 eV for Gd whereas Tb*4f* states, as a result of multiplet splitting, present several lines located at −2.5 eV (^8^*S*_7/2_), −7.4 eV, −9.1 eV, and −10.2 eV [34]. In the case of the compound under investigation, all majority spin *4f* contributions overlap to form one wide band in the range from −11 eV to −6 eV. The shape of the band near the Fermi level is dominated by Co*3d* states and its width is about 4 eV. This energy region also contains contributions of R*5d* and Tb^8^*S*_7/2_ states, however, they are hardly visible on the XPS spectrum since they are obscured by Co*3d* states.

**Figure 10:**
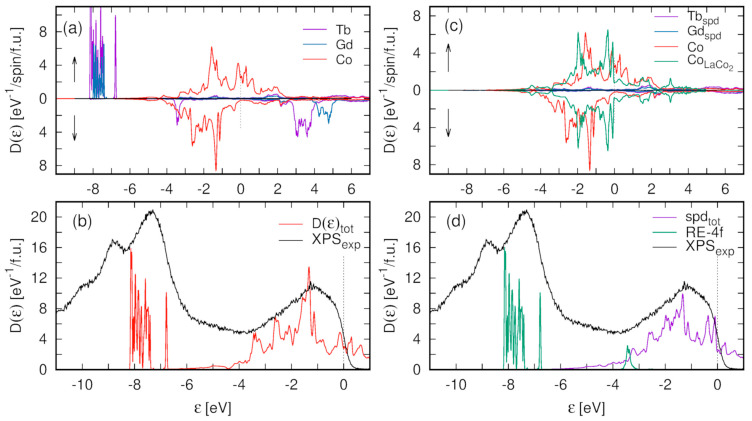
The density of states (DOS) of the Gd_0.375_Tb_0.625_Co_2_ calculated within the PBEsol + SO + U method. (**a**)—spin-resolved, partial atomic contributions, (**b**)—total DOS compared with the scaled XPS spectrum, (**c**)—spin-resolved, partial atomic *spd* DOS in Gd_0.375_Tb_0.625_Co_2_ compared with the *spd* DOS of paramagnetic LaCo_2_ compound; (**d**)—Gd + Tb 4f DOS combined with the simulated mixture of *spd* DOS (60%—Gd_0.375_Tb_0.625_Co_2_ + 40%—LaCo_2_) compared with the scaled XPS spectrum.

Figure 10 shows the calculated partial atomic, spin-resolved density of states (Figure 10a), and the total density of states (DOS) compared with the scaled XPS spectrum (Figure 10b).

The multiplet structure of Tb*4f* states, as the many-body effect, is not accounted for within the single-particle DFT calculations. Nevertheless, the GGA calculations with the Coulomb correlation corrections for Gd*4f* and Tb*4f* states reproduce the observed position of the *4f* state’s manifold.

A significant discrepancy between the shape of the calculated DOS function and the XPS spectrum can be observed just below the Fermi energy, up to 1 eV of BE (Figure 10b). In this energy range, the XPS spectrum shows an evident bump while in the DOS function, a relatively deep valley occurs. The band structure of Co*3d* states in Gd_0.4_Tb_0.6_Co_2_, shown in Figure 10a, is very similar to the results of ab initio calculations reported for TbCo_2_ [20] and GdCo_2_ [35] Laves phases. It comprises two peaks separated by a valley of reduced DOS. The Fermi level is situated at the bottom edge of the upper peak of majority spin Co*3d* states (polarized toward the R magnetic moments) while the minority spin bands are almost fully occupied and located below the Fermi energy. In effect, there are no states which would be responsible for the formation of the bump in the XPS spectrum of the Gd_0.4_Tb_0.6_Co_2_ compound just below E_F_ (Figure 10b). We suppose that the discrepancy between the calculated structure of the valence band and the XPS spectrum near the Fermi level is related to the thermodynamic conditions under which the experiment was conducted. It is worth noting that the total DOS, presented in Figure 10b, is the ground state property, while the XPS spectrum is taken near the Curie temperature, where the magnetic system of the compound is highly unstable. To explain the discrepancy, it is important to note that the magnetic properties of the investigated compound are the result of interplay of two magnetic subsystems: a system of persistent, localized magnetic moments of R ions (*μ_R_*) and a system of local magnetic moments of Co ions (*μ_Co_*) derived from spin-polarized Co*3d* itinerant states due to the exchange interaction with the R*4f* shell. In the vicinity of T_C_, because of strong thermal fluctuations of the R magnetic moments, the local magnetic field exerted by R magnetic subsystem on Co*3d* states may decrease below the critical value, which may imply a reduction of local spin polarization of Co*3d* states. Thus, the XPS spectrum may collect excitations of electrons from Co*3d* states with varying degrees of exchange splitting. We argue, that upon decreasing spin polarization and related exchange splitting of Co*3d* bands, the mutual shift of Co*3d* spin-polarized bands would result in filling the valley separating the peaks of the DOS just below E_F_. The effect may explain the occurrence of the bump observed in the XPS spectrum of Gd_0.4_Tb_0.6_Co_2_ in this energy range.

To check our conjecture, we combine the spin-polarized Co*3d* DOS of Gd_0.4_Tb_0.6_Co_2_ with the corresponding DOS calculated for isostructural LaCo_2_ compound in the paramagnetic state. Both Co*3d* DOS contributions, spin-polarized (in Gd_0.4_Tb_0.6_Co_2_), and paramagnetic (in LaCo_2_) are depicted in Figure 10c. Figure 10d compares the XPS spectrum of Gd_0.4_Tb_0.6_Co_2_ with a simulated mixture of fully spin-polarized (60%) and paramagnetic (40%) Co-*spd* DOS. It is noteworthy that the valley of reduced DOS, present in Figure 10b, is filled partially by non-spin-polarized Co*3d* states, which contribution to the XPS spectrum would result in the bump just below the Fermi level. Figure 10d shows a mixture of DOS with two limiting values of Co*3d* states exchange splitting. The variable magnitude of exchange splitting of Co*3d* states and the related shift of oppositely polarized Co*3d* bands may lead to a more complete filling of the valley of reduced DOS.

**Figure 11:**
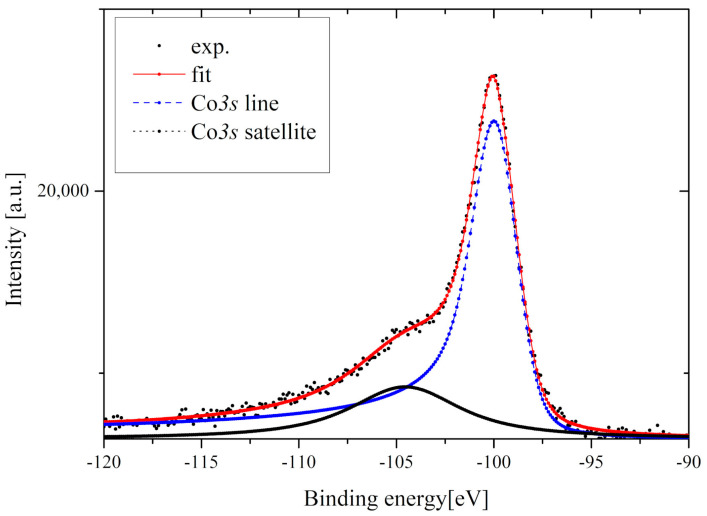
The Co*3s* multiplet splitting for the Gd_0.4_Tb_0.6_Co_2_.

Figure 11 presents the Co*3s* line of the Gd_0.4_Tb_0.6_Co_2_ compound. Analyzing the Co*3s* multiplet splitting one can derive information about the magnetic moment of the cobalt atom. The multiplet splitting of the *3s* spectra of 3*d*-metals is the result of the exchange interaction between unfilled *3d* and ionized *3s* shells. In effect, two final states are observed and the intensities of these peaks satisfy the equation [36]:

(11) $$\;\frac{I_{1}}{I_{2}}=\frac{S+1}{S}$$

where *S* is the spin of the unfilled *3d* shell while *I*_1_ and *I*_2_ are the intensities of the main and the satellite *3s* lines, respectively. The magnetic moment of the Co atom can be estimated using:

(12) $$\;\mu_{Co}=2\mu_{B}\sqrt{S\left(S+1\right)}$$

To obtain the I_1_/I_2_ ratio, necessary to calculate the $\;\mu_{Co}$, the Co*3s* line (Figure 11) was fitted using the Doniach–Sunjić line shape [37]. The background of the spectrum was evaluated using the Touggard function and subtracted [38]. Using the above equations, we obtained the value of the Co magnetic moment equal to 0.95 *μ*_B_ ± 0.05 *μ*_B_ which is slightly lower than the value obtained from the magnetic measurements.

## 5. Conclusions

We synthesized a single-phase polycrystalline Gd_0.4_Tb_0.6_Co_2_ intermetallic compound with an MgCu_2_ type of structure. Magnetic measurements carried out over a wide temperature range confirmed that the magnetic phase transition for this compound is near room temperature (T_C_ = 300.7 K). Both Arrott plots and Landau’s coefficients indicated that, in the investigated compound, we are dealing with the second-order phase transition. The value of the maximum entropy change |Δ*S_M_^max^*| obtained under magnetic field changes *μ*_0_*H* of 7 T is equal to 5.23 J/kgK, which results in the RCP and RC parameters equal to 298.17 and 259.40 J/kg, respectively. The values are moderate compared to some other materials, however, they occur near room temperature, which is crucial for potential applications. The hysteresis loops, at all measured temperatures, showed minimal losses, which is desirable for practical applications in magnetic refrigeration. The value of the magnetic moment of Co determined from the saturation magnetization is comparable with those estimated based on the Co*3s* XPS spectrum, evaluated with the use of MFT analysis and calculated through the ab initio method. The values of exchange coupling parameters *J_RR_*, *J_RCo_*, and *J_CoCo_* obtained from MFT calculations have shown that the strongest interaction occurs between Co-Co spins and the weakest between R-R ones.

The shape of the valence band near the Fermi level is dominated by Co*3d* states. Ab initio calculations showed that R*5d* states also contribute slightly to this energy range. Our photo-emission measurements, combined with the band structure calculations, indicate that near T_C,_ the XPS spectrum collects excitations of electrons from Co*3d* states with different values of exchange splitting. This explains the discrepancy between the measured spectrum and the calculated density of states near the Fermi level.

Summarizing, we have shown that the Gd_0.4_Tb_0.6_Co_2_ compound is a material with minimal hysteresis losses and reasonable RCP values at room temperature, which qualifies it for use in magnetic refrigerators. Furthermore, based on the results of our extended study of magnetic properties (which was completed for the first time by electronic structure investigations) we propose that the Gd_0.4_Tb_0.6_Co_2_ compound may provide a promising basis for a new family of multi-component compounds with enhanced MCE properties.
